# Etiologies and long-term outcome of pediatric hemophagocytic lymphohistiocytosis and macrophage activation syndrome in Taiwan: a single-center retrospective study

**DOI:** 10.3389/fimmu.2025.1596113

**Published:** 2025-07-09

**Authors:** Ching-Yu Wang, Jyh-Hong Lee, Ni-Chung Lee, Ya-Chiao Hu, Hsiu-Hao Chang, Li-Chieh Wang, Yu-Tsan Lin, Yao-Hsu Yang, Bor-Luen Chiang, Hsin-Hui Yu

**Affiliations:** ^1^ Department of Pediatrics, National Taiwan University Hospital Yunlin Branch, Yunlin County, Taiwan; ^2^ Department of Pediatrics, National Taiwan University Children’s Hospital, Taipei, Taiwan; ^3^ Department of Medical Genetics, National Taiwan University Hospital, Taipei, Taiwan; ^4^ Department of Medical Research, National Taiwan University Hospital, Taipei, Taiwan; ^5^ Genome and Systems Biology Degree Program, College of Life Science, National Taiwan University, Taipei, Taiwan

**Keywords:** macrophage activation syndrome, hemophagocytic lymphohistiocytosis, etiology, outcomes, pediatric

## Abstract

Hemophagocytic lymphohistiocytosis (HLH) and macrophage activation syndrome (MAS) are life-threatening hyperinflammatory conditions. Primary HLH is caused by genetic mutations associated with defective cytotoxicity, while secondary HLH is triggered by various factors, including infection-associated HLH (IAHS), rheumatic diseases-associated HLH (MAS), or malignancy-associated HLH (M-HLH). We retrospectively reviewed the medical records of patients younger than 20 years of age with physician-diagnosed HLH or MAS between January 2005 and July 2022 in a large medical center in Taiwan. Seven patients were prospectively enrolled since Jan 2019. Clinical and laboratory features, treatments rendered, and outcomes of patients with HLH/MAS were analyzed. Fifty-two patients with HLH/MAS were included in this study and classified as follows: 21 (40.4%) with IAHS, 20 (38.5%) with MAS, 5 (9.6%) with M-HLH, 4 (7.7%) with primary HLH, and 2 (3.8%) with unclassified HLH (U-HLH). The median age of diagnosis for all patients was 9.04 years, while it ranged between 5.12 (for primary HLH) to 16.03 (for M-HLH) years. Two-year probabilities of survival of each group of HLH/MAS were 100%, 85.7%, 65.63%, 25%, and 20% for patients with U-HLH, IAHS, MAS, primary HLH, and M-HLH, respectively (log-rank, P =0.0018). The five-year probability of survival was 65.63% for patients with MAS. M-HLH and ICU admission were significantly associated with mortality. Infections and rheumatic diseases are the main triggers or conditions associated with pediatric HLH/MAS, whereas malignancy is an important etiology among adolescents.

## 1 Introduction

Hemophagocytic lymphohistiocytosis (HLH) is a severe systemic hyper-inflammation syndrome characterized by fever, cytopenia, hepatosplenomegaly, and elevation of inflammatory and T-cell activation markers, including ferritin and soluble CD25 (sCD25)/IL-2 receptor (sIL-2R) (1). Primary or genetic HLH is caused by inborn immune defects. Approximately 4.1–22% of patients with HLH have genetic mutations associated with cytotoxicity defects or immune dysregulation (2–5). On the other hand, secondary HLH indicates that HLH is induced by environmental factors, including infection-associated HLH (IAHS); rheumatic diseases-associated HLH, also known as macrophage activation syndrome (MAS); malignancy-associated HLH (M-HLH); or without identifiable trigger factors, also known as unclassified HLH (U-HLH) (1, 6, 7).

HLH encompasses a wide range of immune-dysregulated conditions characterized by cytotoxic defects in natural killer (NK) cells, cytotoxic CD8^+^ T cells, or inflammasome regulation. A prolonged contact between lymphocytes deficient in cytotoxic functions and target cells leads to overproduction of pro-inflammatory cytokines from hyperactive macrophages (1). Antigen-presenting cells continue to stimulate activation and proliferation of T cells and form a vicious cycle of lymphohistiocytic proliferation and hypercytokinemia, ultimately leading to widespread hyperinflammation, hemophagocytosis, and tissue damage (1, 8–10).

Timely diagnosis of HLH is important as early intervention and treatment can improve the survival rates (11, 12). However, the diagnosis of HLH in the early stages remains a challenge due to nonspecific and heterogeneous presentations. Moreover, diagnosis is heavily dependent on clinician’s early awareness and judicious judgment (13). The most commonly used diagnostic criteria for HLH are the HLH-2004 criteria (≥five out of eight criteria fulfilment) or hemophagocytic syndrome diagnostic score (HScore) ≥ 169 that corresponds to a sensitivity of 100% and specificity of 80% in children (14–16).

Epstein-Barr virus (EBV) serves as an important trigger of HLH. In a large-scale study conducted in Taiwan, the seroprevalence of EBV was 52.8% in children aged 2 years, 88.7% in those aged 5–7 years, and 93% in those aged 14–16 years. These rates were comparable to the reported seroprevalence of 87–100% among adolescents in other Asian countries but were higher than the 56–65% reported in Europe and North America (17). HLH is frequently associated with EBV infections involving T or NK cells in East Asian countries, and, to a lesser extent, in a few Hispanic patients. This form of HLH follows a more aggressive clinical course and shows limited responsiveness to conventional B-cell-targeted therapies (18–20). In contrast, EBV-associated HLH in Western populations more commonly involves B cells and is often managed with rituximab (21).

The therapeutic approach of HLH/MAS should be tailored based on the underlying etiology or triggering factors. Nevertheless, selecting appropriate immunomodulatory or targeted therapies for HLH/MAS remains challenging. Few observational studies have compared pediatric patients with HLH/MAS across different subgroups (22). Our study aimed to summarize and analyze the clinical or laboratory characteristics at diagnosis, treatment approaches, and survival outcomes according to different pathogenic backgrounds and/or triggers at a largest medical center in Taiwan. Additionally, we aimed to identify prognostic factors for survival in children with different subgroups of HLH/MAS.

## 2 Methods

### 2.1 Study design

We retrospectively reviewed the medical records of 57 patients (aged < 20 years) suspected of having HLH or MAS between January 2005 and July 2022 at the National Taiwan University Children’s Hospital, a tertiary referral medical center in Taiwan. Seven patients were prospectively enrolled since Jan 2019, and healthy controls were recruited for immunological assays.

### 2.2 Inclusion and exclusion criteria

**Figure 1:**
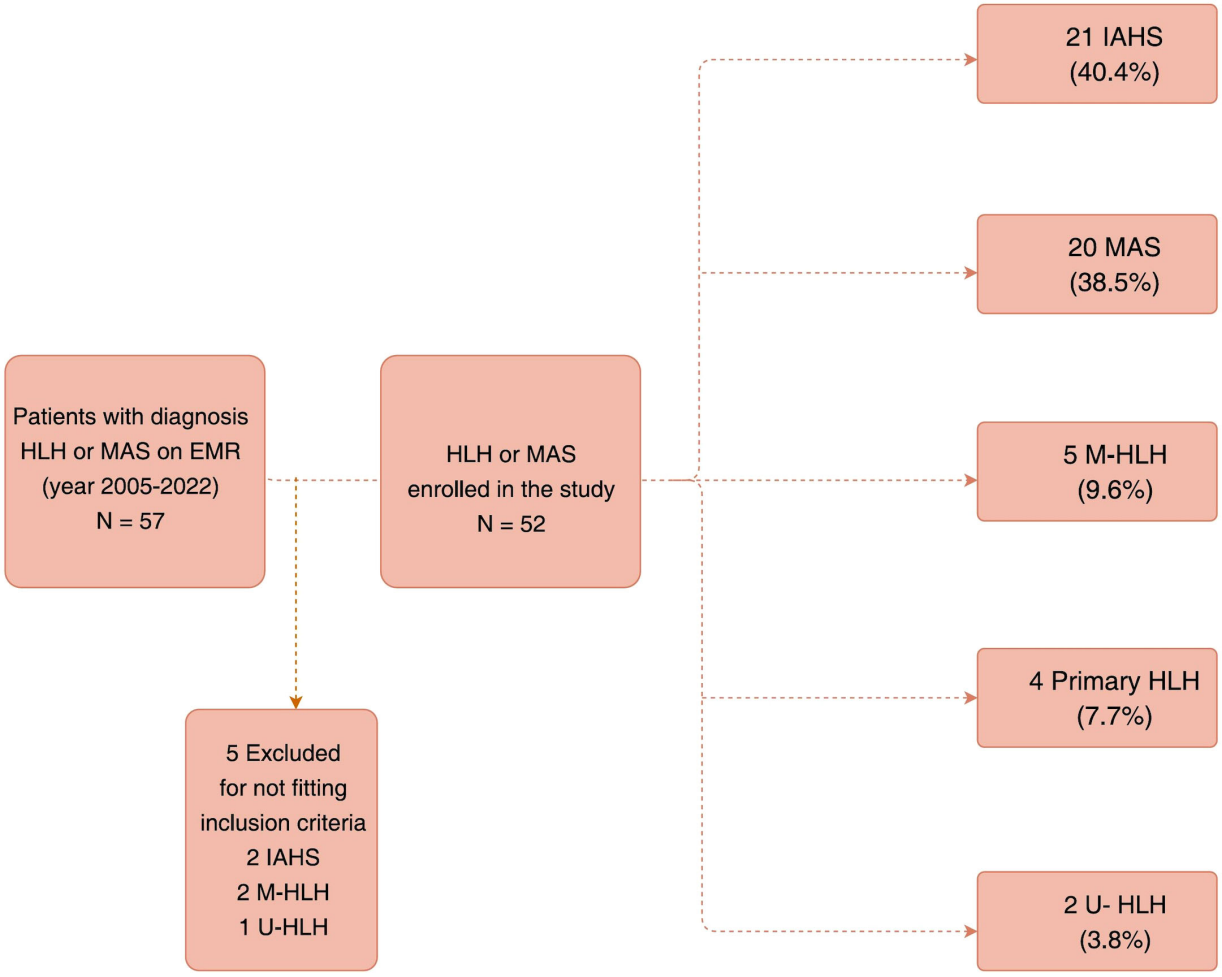
Flow chart demonstrating the selection of study participants.

Patients with HLH who fulfilled five of the eight HLH-2004 criteria or had a genetic diagnosis of primary HLH were included in this study (14). Two patients with IAHS who fulfilled four HLH-2004 criteria were also included considering no alternative diagnosis and the necessity of prompt HLH treatment based on the clinician’s decision. Patients with MAS fulfilled the 2016 classification criteria for MAS complicating systemic juvenile idiopathic arthritis (sJIA) or juvenile systemic lupus erythematosus (SLE) (23, 24). Patients with other rheumatic diseases (Kawasaki disease, Kikuchi disease, and undifferentiated connective tissue disease (UCTD) or syndrome of undifferentiated recurrent fever (SURF) met the aforementioned criteria for MAS (23–26). After excluding five patients who fulfilled only three of the HLH-2004 criteria, 52 patients were finally included in this study (**Figure 1**).

We further classified these patients into five groups based on their underlying etiologies: IAHS, MAS, M-HLH, primary HLH, and U-HLH. IAHS was defined as HLH with preceding clinical features and appropriate treatment responses suggestive of an infectious trigger (with or without an identified pathogen), or clinical features consistent with infection and a confirmed pathogen. Primary HLH was defined as HLH occurring in patients with underlying genetic defects affecting cytotoxic pathways or associated with Inborn Errors of Immunity. One patient, who presented with typical clinical features of Chediak-Higashi syndrome (CHS) and recurrent HLH but lacked a confirmed genetic diagnosis, was included in the study based on fulfillment of the clinical diagnostic criteria for primary immunodeficiency (PID) as defined by the ESID Registry. Detailed medical records, clinical and laboratory data, treatments rendered, and outcomes were collected and retrospectively analyzed.

### 2.3 Ethical considerations

The requirement for informed consent was waived for patients who underwent a medical record review and anonymous clinical data analysis during the retrospective part of the study. Informed consent was obtained from all the patients for the prospective study. This study was approved by the Institutional Research Ethics Committee (approval numbers: 201812007RIND, 201905003RINA, 202006103RINC) and conducted in accordance with the tenets of Declaration of Helsinki.

### 2.4 Genetic analysis

Genetic variations in primary HLH were investigated using Sanger sequencing or whole-exome sequencing (WES). The decision to undertake WES was based on an individualized shared decision-making process involving the patient, their parents, and the healthcare team. The principal criteria for recommending patients for WES included the presence of clinical or laboratory features suggesting primary HLH, underlying inborn errors of immunity, or refractory or recurrent HLH.

### 2.5 Cytotoxicity peripheral blood mononuclear cells assay of NK cells

NK cell cytotoxicity assays were performed in selected patients prior to the initiation of immunomodulatory therapy or after clinical condition stabilized after early management of HLH. Peripheral blood mononuclear cells (PBMCs) obtained from the patients and controls were co-cultured with K562 cells for 4 h. PKH26 Green, a green fluorescent dye, was used to label NK target cells. After incubating PBMCs with different effector/target (E/T) ratios (PBMC:K562 = 100:1, followed sequentially by 50:1, 25:1, 10:1, and 5:1), total cell suspensions were stained using propidium iodide (PI) and flow cytometry analysis was performed. The percentage of PKH26^+^ PI^+^ cells in total number of PKH^+^ cells was used to evaluate NK cell cytotoxicity. NK cytotoxicity in more than 10% of the target cells was defined as normal because NK cytotoxicity ranged between 10.1%-65.7% in healthy controls.

### 2.6 Plasma cytokine and biomarkers

Plasma cytokine analyses were performed during acute stage of HLH. Plasma levels of IL-18, MIG/CXCL9, and soluble CD25 (sCD25) in patients and controls were measured using enzyme-linked immunosorbent assay (ELISA) (R&D, Minneapolis, MN, USA).

### 2.7 Recurrence or relapse of HLH

A relapse of HLH was defined as the reappearance of disease activity after achieving clinical remission but while still undergoing treatment. Recurrence of HLH was defined as a new episode occurring after the successful completion of therapy.

### 2.8 Statistical analysis

Descriptive statistics were expressed as median with interquartile range (IQR) or mean ± standard deviation (SD) for continuous variables and as number (percentage) for nominal variables. For categorical variables, the differences between groups were compared using Fisher’s exact test or Chi-square test. Non-parametric tests were used for the statistical analysis of small sample size. Continuous variables between two groups were compared using the Mann–Whitney U test, while those among more than two groups were analyzed using the Kruskal–Wallis test. Upon observing a statistically significant result from the Kruskal–Wallis test, *post hoc* pairwise comparisons were performed using the Mann–Whitney U test. Logistic regression model was used to calculate the odds ratio (OR) and 95% confidence intervals (CI) for the association between HLH/MAS subtypes and mortality. Sex, diagnostic age of HLH/MAS, EBV infections, and ICU admission were also included in this model as potential confounders based on clinical relevance (such as EBV infection) or univariate significance. Survival was analyzed using Kaplan-Meier method, and log-rank tests were performed for comparison. All data were analyzed using GraphPad Prism (GraphPad Software, San Diego, CA, USA) and SAS software (version 9.4; SAS Institute, Inc., Cary, NC, USA). A two-tailed p-value of less than 0.05 was considered statistically significant.

## 3 Results

### 3.1 Patient characteristics

**Figure 2:**
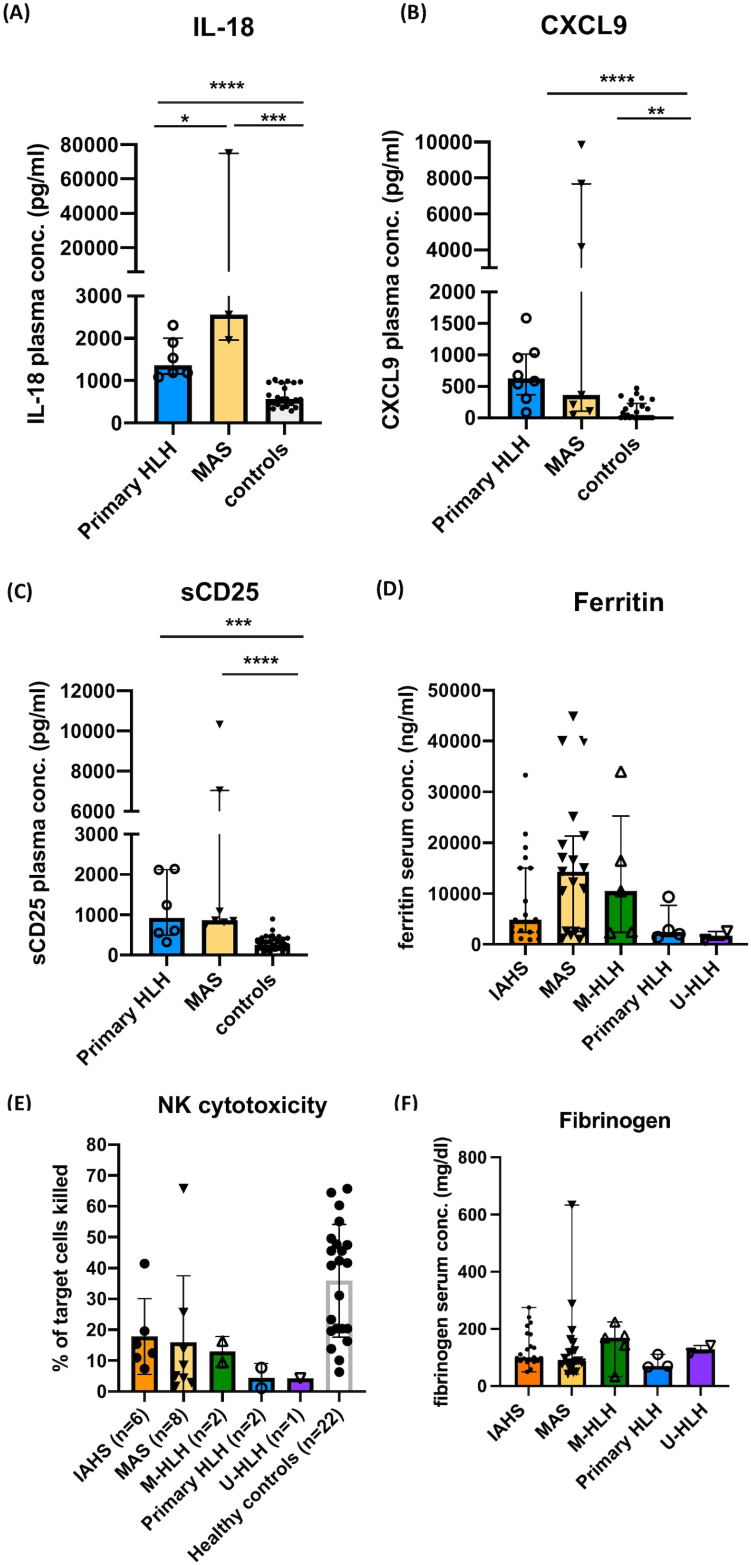
Immunological evaluations performed in this study. Plasma levels of IL-18, MIG/CXCL9, and soluble CD25 (sCD25) in the acute stage of patients with primary HLH, MAS, and controls **(A–C)**. Serum levels of ferritin in the acute stage of patients with HLH/MAS, normal range: 21.81-274.66 (male) and 4.63-204.0 ng/mL (female) **(D)**. NK cytotoxicity (% of target cells killed) in different groups of patients and controls **(E)**. Serum levels of fibrinogen in the acute stage of patients with HLH/MAS **(F)**. Data are expressed as median with interquartile range. *P <0.05, **P<0.01, ***P<0.001, **** *P*<0.0001 by Mann-Whitney U test following statistically significant result from the Kruskal–Wallis test.

Among 52 patients with HLH/MAS (36.5% males), the median age of diagnosis was 9.04 (IQR: 3.7–14.78) years (**Table 1**). There were no statistically significant differences in the diagnostic age among the different HLH subgroups (**Supplementary Figure S1**). The patients were further classified as follows: 21 (40.4%) with IAHS, 20 (38.5%) with MAS, 5 (9.6%) with M-HLH, 4 (7.7%) with primary HLH, and 2 (3.8%) with U-HLH (**Figure 1**). Of note, two patients were initially considered as EBV-associated IAHS but were later confirmed as having T-/NK-cell lymphoma by a series work-up due to recurrence of HLH within 30 days, and were therefore ultimately classified as having M-HLH. Fever and hyperferritinemia (94.2%) were the most common presentations of patients with HLH (**Table 1** and **Figure 2D**). Splenomegaly was present in 40% of patients with MAS, which was significantly lower than that in the other groups (P=0.0185). CNS involvement was observed in 9.5% of patients with IAHS, which was significantly lower than that in the other HLH groups (**Table 1**). Systemic inflammation manifestations such as serositis (75.0%), lung infiltrates (67.3%), lymphadenopathy (57.7%), hepatomegaly (55.8%), acute kidney injury (48.1%), bleeding (40.4%), and skin rashes (36.5%) were commonly observed (**Supplementary Table S1**). The skin rashes were maculopapular rash or urticaria-like, involving the face, trunk, or extremities, and rashes subsided after treatment for MAS or HLH.

**Table 1:** Demographic and clinical features of patients with HLH/MAS.

Subgroups of HLH/Clinical features	All (n=52)	IAHS (n=21)	MAS (n=20)	M-HLH (n=5)	Primary HLH (n=4)	U-HLH (n=2)
Male (n (%))	19(36.54%)	9(42.86%)	3 (15%)	3 (60%)	2 (50%)	2 (100%)
Age at onset (yrs)	8.95 (3.65-14.73)	6.19 (2.98-13.52)	11.13 (3.76-15.27)	15.99 (13.02-17.71)	5.08 (4.32-13.68)	8.19 (3.55-12.83)
Diagnostic age (yrs)	9.04 (3.7-14.78)	6.2 (3.02-13.58)	11.21 (3.87-16.12)	16.03 (13.08-17.75)	5.12 (4.51-13.7)	8.23 (3.55-12.9)
Interval from onset of fever to diagnosis of HLH/MAS (days)*	14.43 (8.01-27.85)	12.05 (7.47-23.93)	23.92 (9.29-50.23)	13.15 (11.98-19.91)	18.08 (6.21-70.21)	17.42 (9.86-24.98)
Follow-up time (yrs)	1.94 (0.44-4.71)	2.14 (0.45-5.52)	1.88 (0.30-4.82)	0.55 (0.20-1.89)	1.18 (0.31-1.93)	6.01 (4.89-7.12)
Clinical and laboratory features						
Fever	49 (94.2%)	21 (100%)	17 (85%)	5 (100%)	4 (100%)	2 (100%)
Splenomegaly	34 (65.4%)	15 (71.4%)	8 (40%)	5 (100%)	4 (100%)	2 (100%)
Cytopenia	39 (75.0%)	16 (76.2%)	13 (65%)	5 (100%)	3 (75%)	2 (100%)
Hypertriglyceridemia and/or hypofibrinogenemia	45 (86.5%)	19 (90.5%)	19 (95%)	4 (80%)	2 (50%)	1 (50%)
Hemophagocytosis	46 (88.5%)	18 (85.7%)	18 (90%)	4 (80%)	4 (100%)	2 (100%))
Hyperferritinemia	49 (94.2%)	19 (90.5%)	20 (100%)	4 (80%)	4 (100%)	2 (100%)
CNS involvement	21 (40.4%)	2 (9.5%)	12 (60%)	2 (40%)	3 (75%)	2 (100%)
From fever to first treatment of HLH (median days)	12 (6.0-23.0) N=46	10.5 (7.5-16.3) N=18	18 (7.0-25.5) N=17	15 (6.0-20.0) N=5	6.5 (4.5-65.5) N=4	5.5 (2.0-9.0) N=2
From fever to steroid use	15 (9.0-25.0) N=45	15 (9-19.5) N=17	22 (8.5-28.5) N=17	15 (6.5-20.0) N=5	20.5 (9.5-71.5) N=4	8.5 (6-11.0) N=2
Mortality	16 (30.8%)	3 (14.3%)	6 (30%)	4 (80%)	3 (75%)	0 (0%)

Data are expressed as medians (interquartile ranges) or number (percentages).

CNS, central nervous system; IAHS, infection-associated hemophagocytic syndrome; MAS, macrophage activation syndrome; M-HLH, malignancy-associated HLH; HLH, hemophagocytic lymphohistiocytosis; U-HLH, unclassified HLH.

*Confirm the diagnosis of HLH status, not final diagnosis of underlying etiologies/subtypes classification.

Among all patients, a total of 40 cases were identified where infection was considered a primary or contributing trigger for HLH/MAS. Of these, 19 cases were associated with EBV, 3 cases with parvovirus, 1 case with adenovirus, and 17 cases involved infections in which the causative pathogen could not be identified but had preceding clinical features suggestive of infections. EBV was identified as the leading pathogen (61.9%) in patients with IAHS, followed by Parvovirus (9.5%). Sixteen of 52 (30.8%) patients had EBV-associated diseases. Among these 17 patients with unidentified pathogens, 8 (47.1%) presented with clinical features consistent with pneumonia or bronchopneumonia, 5 (29.4%) with upper respiratory tract infections including pharyngitis and tonsillitis, 2 with acute gastroenteritis, 1 with a deep neck infection, and 1 with typhlitis. Twenty patients with MAS had underlying rheumatic diseases, such as systemic lupus erythematosus (SLE) (5, 25%), sJIA (5, 25%), Kikuchi disease (2, 10%), and Kawasaki disease (1, 5%). Notably, seven (35%) patients with MAS were diagnosed with UCTD or SURF.

Patients with M-HLH included NK/T-cell lymphoma (N=2), hepatosplenic T-cell lymphoma with leukemic transformation (N=1), acute lymphoblastic leukemia (ALL) (N=1), and acute myeloid leukemia (AML) (N=1). Notably, two patients initially presented with EBV-associated HLH but were later diagnosed with M-HLH after an extensive diagnostic workup was performed to confirm NK/T-cell lymphoma. This included positron emission tomography (PET) and a lymph node biopsy for one patient with inguinal lymphadenopathy, and repeated cytological analysis for another patient with pleural effusion unresponsive to intravenous immunoglobulin (IVIG) and corticosteroid treatment. Unfortunately, although both patients subsequently received treatment targeting their malignancies, they expired before undergoing hematopoietic stem cell transplantation (HSCT).

Two patients were diagnosed by Sanger sequencing, including one four-year-old female patient with familial HLH due to *UNC13D* gene homozygous c.2448-13G>A splice mutation, resulting in a frameshift effect (27). and one five-year-old female patient with Chediak-Higashi syndrome. The genetic variants of *LYST* gene for Chediak-Higashi syndrome was no found. Two patients were diagnosed by WES, included a five-year-old male patient with X-linked lymphoproliferative disease type 1 due to *SAP/SH2D1A* gene c.80G>A (p.Gly27Asp) pathogenic mutation, and a 16.5-year-old male patient with XLP-2 due to *X-linked inhibitor of apoptosis protein (XIAP)/BIRC4* gene c.421_422del (p.Leu141fs) pathogenic mutation, while his mother and an elder sister were asymptomatic carriers. HSCT was performed for the patient with XLP-2 at the age of 16.9 years with successful engraftment and control of HLH activity.

### 3.2 Laboratory and immunological findings

Patients with primary HLH (N=8) and MAS (N=7) showed significantly elevated plasma levels of IL-18, CXCL9, and sCD25. Plasma levels of IL-18 were significantly elevated in patients with MAS in comparison to those with primary HLH (**Figures 2A–C**). NK cell cytotoxicity assay was performed in 19 (36.5%) patients with HLH/MAS. Among these patients, NK cell cytotoxicity assays were performed before the initiation of immunomodulatory treatment in 47.4% of cases. The remaining patients underwent testing after treatment had already commenced, as immediate intervention was required to manage life-threatening hyperinflammatory activity. The median interval between the initiation of therapy and the performance of the NK cell cytotoxicity assay was 8 days, with an interquartile range of 3.5 to 14 days. Abnormal NK cell cytotoxicity was observed in 10 of 19 (52.6%) patients. Absence of NK cell cytotoxicity was observed in five of 19 (26.3%) patients, which comprised three of eight (37.5%) patients with MAS, one of two (50%) patients with primary HLH, and one (100%) with U-HLH (**Figure 2E**, **Supplementary Table S2**). The median of the lowest fibrinogen levels recorded during the HLH episode was 99.5 mg/dL (interquartile range: 81–160 mg/dL). There were no statistically significant differences in the serum levels of fibrinogen, ferritin, or NK cytotoxicity, between groups (**Figures 2D–F**).

### 3.3 Treatment and outcomes

**Figure 3:**
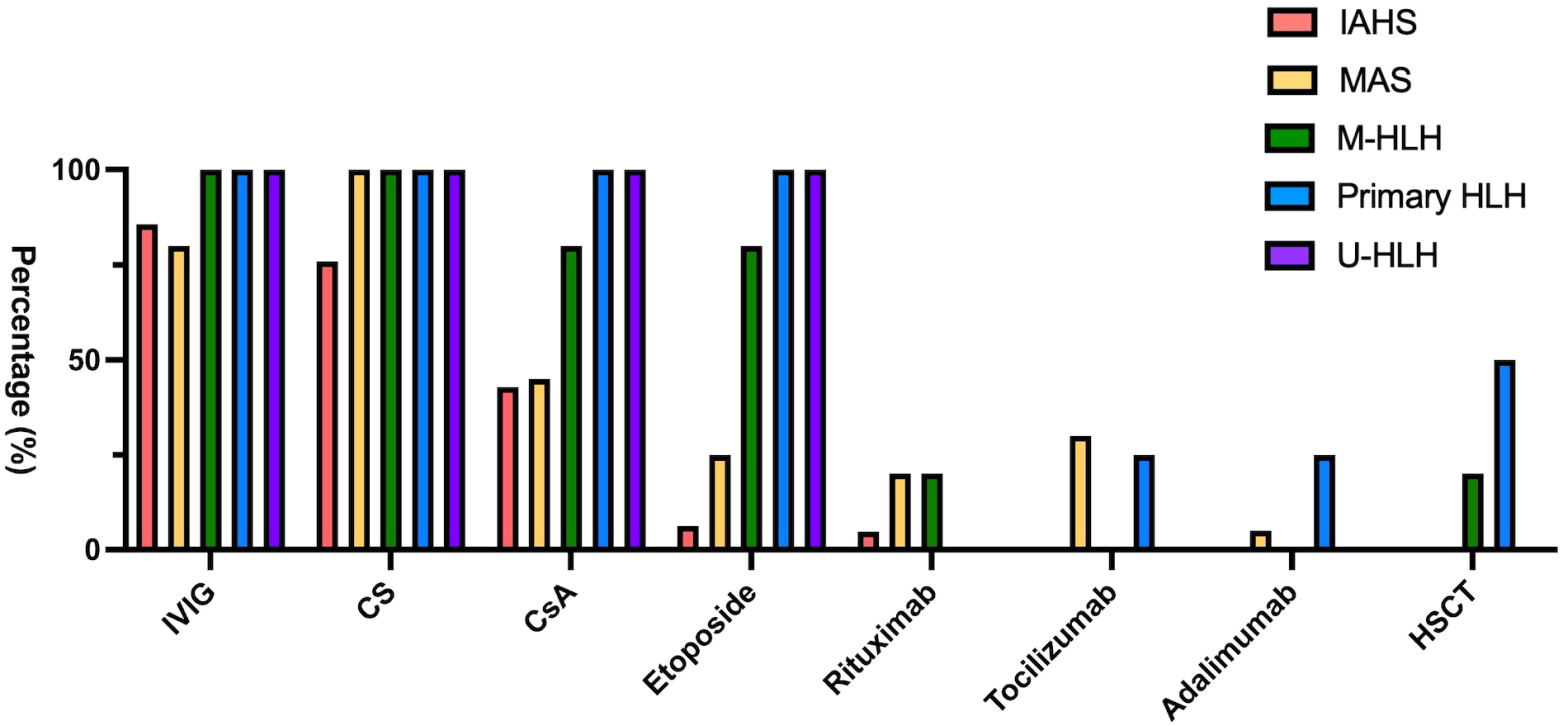
Bar chart demonstrating different treatment modalities rendered to patients with distinct HLH subtypes.

The treatments rendered for HLH or MAS are summarized in **Figure 3**. Majority (94.23%) of the patients were treated in accordance with HLH-2004 protocols or modified HLH-2004 protocols based on disease activity. First-line therapy included high-dose IVIG and corticosteroids, which was followed by cyclosporin and etoposide (**Figure 3**). Approximately half of the IAHS patients were treated with IVIG and/or corticosteroids, while 42.9% and 6.3% of the IAHS patients required cyclosporin and etoposide, respectively, to achieve disease control. Biologics, including tocilizumab (a humanized anti-interleukin-6 receptor monoclonal antibody), rituximab (a chimeric anti-CD20 monoclonal antibody), or adalimumab (a humanized neutralizing anti-tumor necrosis factor monoclonal antibody), were used if the initial therapy failed to control the symptoms of HLH, especially among patients with MAS (**Figure 3**). Rituximab was used in selected cases of EBV-associated HLH or patients with autoimmune diseases, particularly SLE. Tocilizumab and adalimumab were used in patients with JIA or in cases of systemic inflammation characterized by elevated serum interleukin-6 (IL-6) or tumor necrosis factor-alpha (TNF-α) levels, respectively.

One male patient with XIAP deficiency developed an acute HLH flare following HSCT, characterized by markedly elevated serum ferritin levels (up to 33,710 ng/mL) and persistent cytopenia. Due to a partial response to corticosteroids and IVIG, ruxolitinib was subsequently administered, resulting in clinical improvement, resolution of cytopenia, and a significant reduction in both ferritin and sCD25 serum levels.

Thirty-three (63.5%) patients with HLH/MAS required intensive care, including 72.7% shock with fluid resuscitation and/or inotropic agents, 39.4% respiratory failure with ventilator support, 39.4% massive gastrointestinal or pulmonary hemorrhage, 18.2% acute kidney injury requiring renal replacement therapy, 15.1%, acute liver failure and 12.1% status epilepticus. Two patients with M-HLH and two with primary HLH received allogeneic HSCT, but only two patients (one XLP-2 and one ALL) survived for a duration of 1.7 - 3.1 years post-HSCT. Complications of HSCT included severe infections, such as pneumonia or sepsis (100%), reactivation of HLH (75%), and graft-versus-host disease (50%). Pneumonia was the leading cause of death post-HSCT.

**Figure 4:**
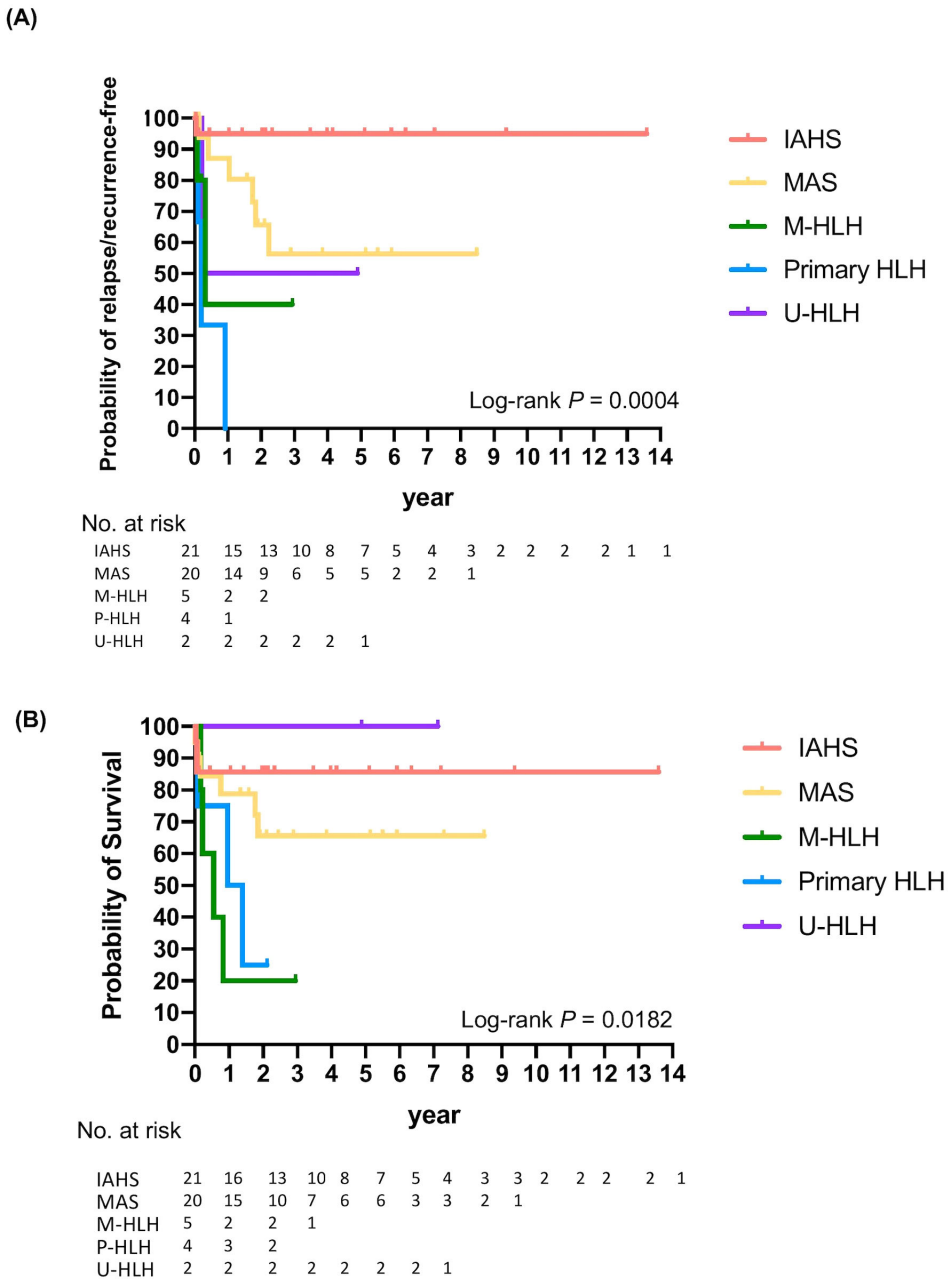
Kaplan-Meier analysis demonstrating relapse or recurrence-free **(A)** and overall survival **(B)** of patients with different subtypes of HLH.

Thirteen (25%) patients with MAS, M-HLH, or primary HLH experienced a relapse or recurrence of HLH (**Figure 4**). The probabilities of relapse or recurrence of HLH were significantly different among the groups (log-rank *P*=0.0004): 100% in primary HLH, 60% in M-HLH, 50% in U-HLH, 13.0% in MAS, and 5% in IAHS one year after the first episode of HLH. Approximately 43.7% patients with MAS experienced recurrence within five years. The relapse or recurrence of HLH occurred in patients with M-HLH and underlying conditions of NK/T-cell lymphoma, hepatosplenic T-cell lymphoma with leukemic transformation, or AML, and in patients with MAS and underlying conditions of sJIA (60% relapse/recurrence rate), UCTD or SURF (38% relapse/recurrence rate), or SLE (33% relapse/recurrence rate) (**Figure 4A**, **Supplementary Table S3**).

Despite aggressive treatment, all-cause mortality rate of HLH/MAS was 30.8% (16/52) in our cohort. The probabilities of survival differed significantly among the groups (log-rank *P* =0.0182). Two-year probabilities of survival were 100%, 85.7%, 65.6%, 25%, and 20% for U-HLH, IAHS, MAS, primary, and malignant HLH, respectively. The five-year probabilities of survival were 100% for U-HLH, 85.7% for IAHS, and 65.6% for MAS (**Figure 4B**, **Supplementary Table S3**).

### 3.4 Prognostic factors

Multivariate logistic regression analysis revealed that M-HLH and ICU admission were significantly associated with mortality with OR (95% CI) of 97.19 (3.05–999.99) and 12.56 (1.26–125.16), respectively (P<0.05), after adjusting for sex, diagnostic age, and EBV infection (**Table 2**). A comparison between pediatric HLH (<12 years at diagnosis) and adolescent HLH (≥12 years at diagnosis) revealed no statistically significant differences in sex distribution, EBV infection rate, underlying etiology, follow-up duration, or mortality (**Supplementary Table S4**).

**Table 2:** Prognostic factors associated with mortality by logistic regression model.

Outcome/Risk factors	Deceased (N=16)	Alive (N=36)	OR (95% CI)	Multi-variate *P* value
Male sex	6 (37.5%)	13 (36.1%)	0.86 (0.15-4.92)	0.862
Diagnostic age	10.76 (4.05-15.89)	7.63 (3.67-14.71)	0.94 (0.82-1.09)	0.424
EBV infection	6 (37.5%)	13 (36.1%)	2.18 (0.23-125.16)	0.500
HLH type^a^				
IAHS	3 (18.75%)	18 (50%)	Ref^a^	
MAS	6 (37.5%)	14 (38.89%)	5.64 (0.48-66.72)	0.170
M-HLH	4 (25%)	1 (2.78%)	97.19 (3.05->999.99)	0.010
Primary HLH	3 (18.75%)	1 (2.78%)	12.73 (0.84-191.87)	0.067
U-HLH	0 (0%)	2 (5.56%)	<0.001 (<0.001->999.99)	0.984
ICU admission	14 (87.5%)	19 (52.78%)	12.56 (1.26-125.16)	0.031

Data are expressed as medians (interquartile ranges) or percentages.

HLH, hemophagocytic lymphohistiocytosis; IAHS, infection-associated hemophagocytic syndrome; MAS, macrophage activation syndrome; M-HLH, malignancy-associated HLH; ICU, intensive care unit.

a IAHS was used as the reference group for multivariate logistic regression.

## 4 Discussion

Our study described the clinical features, underlying diseases or triggers, treatments rendered, and long-term outcomes of pediatric patients with HLH/MAS in Taiwan. Patients with IAHS (40.4%) and MAS (38.5%) comprised the majority of our study population. The distribution of etiology groups in our study differs from that of large-scale studies, which showed that infection (9–75%) and malignancy (26–73%) were the most common triggers or underlying diseases, followed by rheumatic diseases (2–26%) in adult patients with HLH (28, 29). On the other hand, IAHS (9–88%), primary HLH (3–46%), MAS (2–26%), and M-HLH (2–19%) were reported in children with HLH/MAS (2, 29–32).

A better understanding of the pathophysiology of MAS and the development of diagnostic criteria have enhanced its early recognition. However, no single set of criteria is considered sufficient to diagnose HLH/MAS syndrome across all contexts (23, 24, 32, 33). The higher prevalence of MAS in our study might be related to the fact that our institute is a large medical center for children in Taiwan. Approximately 75% of our patients with MAS fulfilled five or more of the HLH-2004 criteria. The unfulfilled criteria were elevated sCD25, low NK cytotoxicity, cytopenia, and splenomegaly, which was compatible with underlying chronic hyperinflammation in patients with MAS. Our results demonstrated that highly elevated serum/plasma levels of IL-18 and ferritin were salient laboratory features of MAS, whereas NK cell cytotoxicity assays lacked diagnostic specificity, ranging from being absent to normal functioning. The marked elevation of IL-18 observed in our cohort was consistent with findings reported by Weiss et al., who demonstrated that IL-18 not only plays a pathogenic role in MAS but also serves as a diagnostic marker (34).

On the other hand, variability in NK cytotoxicity results could be due to multiple factors, such as inherent defects in the patient’s immune function, temporary suppression from steroid or immunosuppressive treatments, the impact of leukopenia at the time of the test, or variability within the control group. To be noted, the level of elevation ferritin, IL-18, CXCL9, and sCD25 in the cohort is relatively modest compared to previously reported values (1). It may be attributed to prior immunosuppressive therapy in some patients at the time of measurement, which may have attenuated the levels of biomarkers. The interpretation of NK cell cytotoxicity and biomarkers results in this study should be approached with caution due to variability in the timing of sample collection relative to the initiation of immunomodulatory therapy.

The onset of primary HLH usually occurs at a young age; however, school-age children and teenagers do not preclude the possibility of a genetic cause of HLH, which was demonstrated in this study. Low or absent NK cytotoxicity function, high sCD25 levels, modest elevation of ferritin levels, and rapid recurrence within six months of the first episode of HLH are clues for primary HLH. Given the improvements in genetic testing and its potentially significant impact on management strategies and prognosis, there is an increasing trend to incorporate genetic surveys into the initial evaluation of HLH (1, 35). Furthermore, in male patients, it is particularly crucial to consider XLP and XIAP deficiency, given the significant variability in the clinical phenotypes of these conditions and the potential need for implementation of HSCT (36, 37).

EBV is reportedly the leading triggering pathogen with a prevalence of 36.3–66.2% among Asian children, which was similar to our findings (2, 5, 30, 31). The mechanisms and the predilection of Asian population for EBV-associated HLH remains unclear. EBV-associated HLH, especially in those developing chronic active Epstein-Barr virus infection, usually relapses or is refractory, and exhibits poor prognosis and high lethality without HSCT (38, 39). Chronic active Epstein-Barr virus infection was suspected in one patient with relapsed/recurrent EBV-related HLH during tapering of the initial monotherapy of corticosteroid. The second course was then controlled with the cyclosporin with more cautious tapering. Importantly, the presence of EBV does not exclude the possibility of other underlying HLH subtypes. Comprehensive evaluation is essential for the early identification of alternative or coexisting causes, particularly in cases of refractory HLH. It has also been reported that the combination of HLH-2004 protocol with biologics targeting cellular reservoirs, such as rituximab or nivolumab (a PD-1 immune checkpoint inhibitor), could improve the outcome of EBV-HLH (21, 38).

Notably, a high proportion of patients in our cohort received IVIG (80-85% in patients with IAHS and MAS, and 100% in patients with M-HLH, primary HLH, and U-HLH) as a part of initial management. Our practice aligns with the 2022 EULAR/ACR recommendations that early administration of IVIG may provide immunomodulation without significant immunosuppression and without impairing malignancy workup (32).

Advances in genetic diagnosis, HSCT, along with therapy for malignancy and autoimmune diseases have facilitated the overall treatment of patients with HLH/MAS (40). It is important to adopt individualized management policies to treat the heterogeneous etiologies of HLH (32). Biologics of anti-IL-1 therapy (anakinra) or anti-interferon-gamma- monoclonal antibodies (emapalumab) were not available in Taiwan (41, 42). In our study, tocilizumab and rituximab were used as biological agents for refractory HLH, especially MAS (43, 44). HSCT is indicated for primary HLH, refractory/recurrent HLH or in certain patients with malignancies, although the post-HSCT survival in patients with primary HLH has been reported to remain suboptimal in recent studies (45, 46). On the other hand, ruxolitinib, a Janus kinase (JAK) 1 and 2 inhibitor that blocks signaling of pro-inflammatory cytokines including IFN-γ and IL-6, has been proven to be effective for the treatment of pediatric HLH (47). Given that patients with XIAP deficiency are likely to be more susceptible to complications such as graft-versus-host disease and post-HSCT HLH (48), our experience of a single patient with XIAP deficiency suggests that ruxolitinib may have a beneficial effect in controlling both hyperinflammation and post-HSCT HLH activity; however, larger studies are needed to confirm its efficacy.

Mortality rate of HLH remained high of 22–59% despite aggressive treatment (2, 3, 11, 28, 29, 49). We found that M-HLH had the highest risk of recurrence or relapse within one year and mortality within two years after the diagnosis of HLH. In our study, the mortality rate of patients with MAS was 30%. The mortality rates of SLE-associated- and sJIA-associated MAS in children varied in previous studies, ranging between 12.5-50% and 0-15.2%, respectively (50–52).

Our study had several strengths, including the accuracy of etiology survey and analysis of long-term outcomes. This study also had a few limitations that need consideration. This was a single-center observational study with a limited sample size, selection bias, and missing data due to retrospective design, which might have limited the generalizability of our findings to a larger population. In addition, the categorization of HLH subgroups in our study may be subject to classification bias, especially since WES was not conducted for all patients with HLH. Our findings should be interpreted cautiously.

Accumulating more cases and experiences would enhance our understanding for rare conditions, such as U-HLH. Nevertheless, the findings of this study offer invaluable information for clinical management of this heterogeneous patient population.

## 5 Conclusion

EBV was identified as the leading pathogen in patients with IAHS. Infections and rheumatic diseases were the primary triggers of pediatric HLH, while M-HLH was an important etiology among adolescents. Elevated serum ferritin and IL-18 levels may be indicative of MAS. M-HLH and ICU admission were significantly poor prognostic factors for survival.

## Data Availability

All datasets are incorporated into the article and its online **Supplementary Material**. The data underlying this article will be shared on reasonable request to the corresponding author.
