# Supplementary material for: Etiologies and long-term outcome of pediatric hemophagocytic lymphohistiocytosis and macrophage activation syndrome in Taiwan: a single-center retrospective study
Source: Front Immunol. 2025 Jul 9;16:1596113. doi: 10.3389/fimmu.2025.1596113 (PMC12284797; doi:10.3389/fimmu.2025.1596113)
Supplement: Supplementary file 1 [file DataSheet1.pdf]

Supplementary Material

Supplementary Table S1. Other clinical features of patients with HLH/MAS

	All (n=52)	IAHS (n=21)	MAS (n=20)	M-HLH (n=5)	Primary HLH (n=4)	U-HLH (n=2)
Serositis	75.0%	76.2%	80%	80%	50%	50%
Lung infiltrates	67.3%	61.9%	75%	80%	75%	0%
Lymphadenopathy	57.7%	47.6%	60%	80%	75%	50%
Hepatomegaly	55.8%	57.1%	40%	80%	75%	100%
Acute kidney injury	48.1%	38.1%	45%	80%	75%	50%
Bleeding	40.4%	33.3%	45%	40%	75%	0%
Rash	36.5%	42.9%	40%	40%	0%	0%

Abbreviations: infection-associated hemophagocytic syndrome, IAHS; macrophage activation syndrome, MAS; malignancy-associated HLH, M-HLH; hemophagocytic lymphohistiocytosis, HLH; unclassified HLH, U-HLH

Supplementary Table S2. NK cell cytotoxicity in patients with HLH/MAS.

Group	N	Normal (≥ 10%)[a]	Low (<10%)[a]	Absent (<5%)[a]
IAHS	6	5/6 (83.3%)	1/6 (16.7%)	0
MAS	8	3/8 (37.5%)	2/8 (25%)	3/8 (37.5%)
M-HLH	2	1/2 (50%)	1/2 (50%)	0
Primary HLH	2	0	1/2 (50%)	1/2 (50%)
U-HLH	1	0	0	1 (100%)

[a]NK cell cytotoxicity function was expressed as percentage of target cells that were killed.

Abbreviations: infection-associated hemophagocytic syndrome, IAHS; macrophage activation syndrome, MAS; malignancy-associated HLH, M-HLH; hemophagocytic lymphohistiocytosis, HLH; unclassified HLH, U-HLH

Supplementary Table S3. Kaplan-Meier estimated probabilities of relapse/recurrence-free survival and survival

	Probability of relapse/recurrence-free			
**Group**	**0.5-yr**	**1-yr**	**2-yr**	**5-yr**
IAHS	95%	95%	95%	95%
MAS	87.05%	87.05%	65.75%	56.35%
M-HLH	40%	40%	40%	-
Primary HLH	33.3%	0%	-	-
U-HLH	50%	50%	50%	50%
	**Probability of Survival**			
**Group**	**0.5-yr**	**1-yr**	**2-yr**	**5-yr**
IAHS	85.71%	85.71%	85.7%	85.7%
MAS	84.38%	78.75%	65.63%	65.63%
M-HLH	60%	20%	20%	-
Primary HLH	75%	50%	25%	-
U-HLH	100%	100%	100%	100%

Abbreviations: infection-associated hemophagocytic syndrome, IAHS; macrophage activation syndrome, MAS; malignancy-associated HLH, M-HLH; hemophagocytic lymphohistiocytosis, HLH; unclassified HLH, U-HLH

Supplementary Table S4. Clinical features in patients with pediatric HLH (<12 years of age at diagnosis) and adolescent HLH (≥ 12 years of age at diagnosis)

	Pediatric HLH (N=32)	Adolescent HLH (N=20)	*P* value
Male sex	11 (34.38%)	8 (40%)	0.682
Diagnostic age	4.055 (2.74-6.43)	15.99 (14.35-17.47)	<0.0001
EBV infection	11 (34.38%)	8 (40%)	0.682
HLH type			0.292
IAHS	15 (46.88%)	6 (30%)	
MAS	12 (37.5%)	8 (40%)	
M-HLH	1 (3.13%)	4 (20%)	
Primary HLH	3 (9.38%)	1 (5%)	
U-HLH	1 (3.13%)	1 (5%)	
Mortality	10 (31.25%)	6 (30%)	0.924
Follo-up time (yr)	1.88 (0.61-5.08)	2.13 (0.20-3.81)	0.771

Data are expressed as medians (interquartile ranges) or percentages

Abbreviations: hemophagocytic lymphohistiocytosis, HLH; infection-associated hemophagocytic syndrome, IAHS; macrophage activation syndrome, MAS; malignancy-associated HLH, M-HLH, unclassified HLH, U-HLH

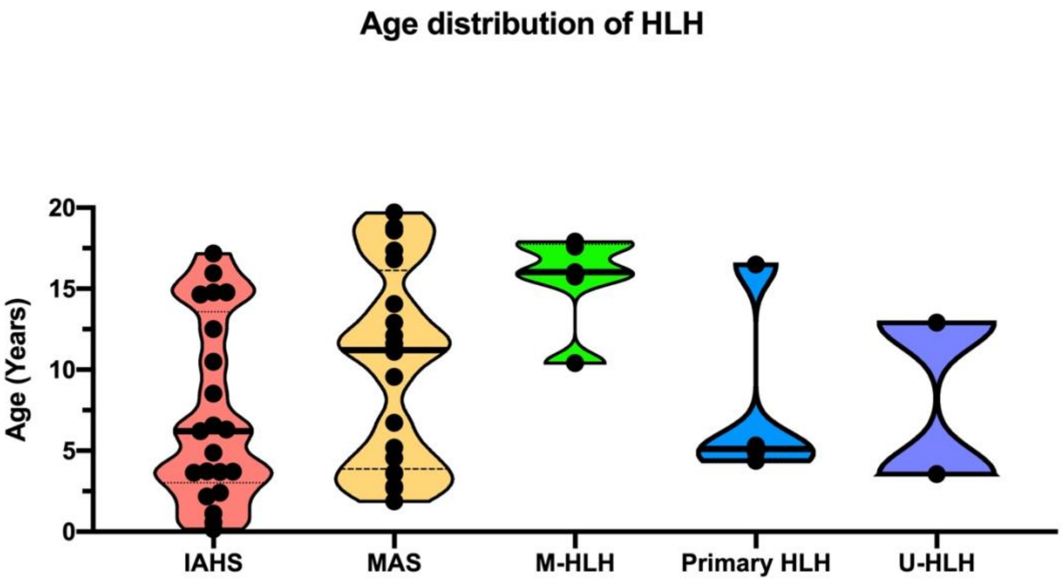

Supplementary Figure S1. Violin plot depicting age at the time of HLH onset (expressed as median with interquartile range).
